# Acute urinary retention in a young man secondary to colonic irrigation: a case report

**DOI:** 10.4076/1752-1947-3-6757

**Published:** 2009-06-11

**Authors:** Omer A Raheem, Ronan M Long, Rowan G Casey, Frank T D'Arcy, Thomas H Lynch

**Affiliations:** 1 Department of Urology, University of Dublin, Trinity College, St James's Hospital, James's Street, Dublin 8, Ireland

## Abstract

**Introduction:**

Autonomic innervation of the bladder is complex and regulated by a hierarchy of mechanisms of the central nervous system. Any dysfunction in these regulatory mechanisms can lead to acute urinary retention.

**Case presentation:**

A 36-year-old Caucasian man presented with acute urinary retention following extensive bowel irrigation. His urinary bladder was decompressed and his normal voiding mechanism was restored thereafter.

**Conclusion:**

We postulate that prolonged anorectal and sigmoid dilatation can stimulate the recto-vesicourethral reflex and lead to acute urinary retention via autonomic dysfunction.

## Introduction

A history of acute urinary retention (AUR) in healthy young men is an infrequent presentation. AUR is defined as an emergency situation characterized by a sudden inability to pass urine and is associated with lower abdominal pain and distention [1].

The aetiology of AUR in young men can be broadly classified into obstructive, neurogenic or pharmacologic [2]. The obstructive causes of AUR may be related to severe prostatitis, stricture or calculi in the urethra [2]. The neurogenic causes of AUR, which are less common, are multiple sclerosis, spinal cord trauma or prolapsed intervertebral disc [2]. Narcotics and alcohol abuse are considered pharmacologic causes of AUR. Other rare causes have also been reported in the published literature [3]-[6].

Immediate management of AUR involves insertion of a urinary catheter to drain the urinary bladder and relieve patient distress. Nevertheless, further urological investigations should be carried out to determine the underlying pathology of such dysfunctional voiding [2].

The manifestation of AUR as a result of bowel abnormalities can be explained partly by the common embryogenesis of the lower urinary and anorectal systems. More importantly, the recto-vesicourethral reflex is the main physiologic reflexive mechanism that harmonizes the defecation and urination functions in the normal physiological state. Specific pathophysiological problems such as prolonged rectal distention can initially disrupt the recto-vesicourethral reflex and ultimately lead to the development of AUR.

## Case presentation

A 36-year-old Caucasian man presented to the emergency department of a university teaching hospital with a 12-hour history of acute lower abdominal pain and distension associated with the inability to pass urine. A detailed clinical history revealed no history of any urological, neurological or sexually transmitted disease. He had neither previous abdominal, spinal or genital trauma, nor substance or alcohol abuse. He also denied any history of constipation. This patient had no previous lower urinary tract symptoms (LUTS). However, extensive bowel irrigation (approximately 5 litres at room temperature over a 5-hour period) was performed due to hygienic purposes prior to his presentation. No rectal pain or discomfort was experienced by the patient during bowel irrigation.

On clinical examination, the patient was in obvious suprapubic discomfort. However, his cardiorespiratory and neurological examinations were normal. A tender suprapubic mass was palpable up to the umbilicus, which is consistent with acute urinary retention. Results of his genital and penile examinations were also normal. His digital rectal examination revealed a small, benign and non-tender prostate. Rectal sphincter tone and peri-anal sensation were normal and the rectum was empty of faeces. A clinical diagnosis of AUR was made and his urinary bladder was decompressed by insertion of 18 FG indwelling Foley catheter which yielded 2000 ml of clear urine.

Results of laboratory haematological studies including full blood count, C-reactive protein, erythrocyte sedimentation rate and random blood sugar were all normal. The biochemical tests showed marked elevation of serum creatinine level 701 µmol/l (normal range 60 to 100 µmol/l) on admission. His urinalysis was normal and without pyuria or haematuria. Bilateral moderate hydroureteronephrosis to the level of the urinary bladder were identified on renal ultrasonography. The patient's serum creatinine level dropped to 153 µmol/l eight hours after urinary catheterization.

The patient was then hospitalized and subsequently underwent full urological and radiological assessment. Post-void residual, uroflowmetry and cystoscopy examinations were performed during the next few days following hospitalization and revealed normal findings. Urodynamic studies were postponed for more than three days and the findings were also normal. Magnetic Resonance Imaging (MRI, Multiple Sclerosis protocol) of his brain and spinal cord were both clear. Serum creatinine level returned to normal in the second day post-admission. Two days later, a repeat renal ultrasonographic examination demonstrated normal sized kidneys and no evidence of urinary obstruction. Prior to patient discharge, he was taught to use clean intermittent self-catheterization (CISC) by a urology nurse specialist. Follow-up assessment two months later showed that the patient emptied his bladder completely and had no LUTS. He used CISC for only 10 days after his discharge from hospital.

## Discussion

This is a very unusual case of AUR in a young man and we inferred that this was related to his bowel irrigation. There is no similar case in the literature. After full urological and neurological assessments, we concluded that colonic and ano-rectal dilation caused by bowel irrigation and distension was the cause of his AUR. Normal micturation was restored in our patient 10 days after presentation.

Although colonic cleansing using different preparations such as phosphate have been safely used in numerous diagnostic and surgical procedures, acute retention of urine has not been reported as a possible side effect [7]. This side effect can partly be explained by the insufflation of rapid and copious amounts of irrigation fluid into the rectum leading to an extensive rectal distention and subsequent AUR.

Innervation of the bladder is complex and regulated by a hierarchy of central nervous system mechanisms, and abnormalities can give rise to acute urinary retention. Bladder function is considered to undergo both storage and voiding phases. In the storage phase, the sympathetic drive (T_10_ -L_2_) exerts an inhibitory effect on the parasympathetic mediated detrusor muscle tone and allows external sphincter (rhabdosphincter) contraction. Detrusor muscle contraction is normally coordinated by the excitatory effect of the parasympathetic nerve (S_2_-S_4_) under the control of the pontine micturition centre in the brainstem (voiding phase).

Miyazato et al. [8], in an experimental animal model, examined the inhibitory role of rectal distention on detrusor contraction in rats. The rectum was inflated by a balloon and the bladder detrusor pressure was then measured cystometrically. This was followed by intrathecal injection of strychnine (a selective glycine receptor antagonist) or bicuculline (GABA receptor antagonist), which blocked the reflex and restored normal detrusor contraction. In this study, it was illustrated that the induced rectal distention, up to 3 cm^3^, decreased bladder contraction. This animal study inferred the existence of an inhibitory recto-vesical reflex, which is modulated by the glycinergic or GABAergic mechanisms in the lumbosacral cord of rats [8].

Shafik et al. [9] subsequently developed a human model based on Miyazato's observation. They identified the presence of the recto-vesicourethral reflex. Fifteen healthy individuals had their rectum anesthetized with a lidocaine solution consisting of 20 ml of 2% lidocaine to block the stretch receptors. The rectum was then distended with a balloon in increments of 50 ml to 300 ml. The vesical and urethral pressures were measured with catheter transducers in response to rectal balloon distension three hours and 20 minutes after lidocaine administration. The procedure was then repeated using rectal administration of normal saline instead of lidocaine. In this study, rectal distention of up to 300 ml was associated with a significant decrease in the intra-vesical pressure (mean pressure: 3.2 ± 0.5 cmH_2_0; range: 1.7 to 4 cmH_2_0), while increasing urethral pressure (mean pressure: 103.2 ± 10.8 cmH_2_0; range: 87 to 126 cmH_2_0) [9].

This reflex physiological process can be explained by rectal distention leading to vesical dilatation and an increase in the urethral sphincter tone mediated via the recto-vesicourethral reflex. Thus the recto-vesicourethral reflex acts independently to harmonize defecation and urination mechanisms in the normal physiological state. If a prolonged rectal distention occurs, the recto-vesicourethral reflex is over-stimulated leading to urinary retention via significantly diminished vesical and increased urethral sphincter pressure. The clinical significance of such reflex may be manifested in patients with severe constipation [9]. Pelvic nerve damage following anorectal surgery can also lead to dysfunctional voiding [10].

Godec previously documented the development of AUR following anal dilatation produced by homosexual activity in four young males [11]. He subsequently designed an experimental human study to evaluate the effect of anal dilatation on bladder dysfunction based on his observations [12]. The bladder pressure was cystometrically measured in five patients with marked urgency and urge incontinence. This demonstrated detrusor overactivity and an average bladder capacity of 86 ml. Anal dilation from 3.5 cm to 5 cm was performed, which increased bladder capacity to an average value of 406 ml on cystometrogram. This demonstrates that mechanical stretch stimulation of the anal region can produce reflex bladder inhibition [12].

## Conclusions

The occurrence of AUR secondary to extensive bowel irrigation in a young man is extremely rare. This case report highlights the importance of considering the possibility of such diagnosis when investigating a young patient with AUR. Adequate and detailed history and clinical examinations are necessary to avoid inappropriate management. Restoration of normal micturation can be achieved subsequently with minimal intervention.

## Abbreviations

AUR: acute urinary retention; CISC: clean intermittent self-catheterization; FG: French gauge; LUTS: lower urinary tract symptoms.

## Consent

Written informed consent was obtained from the patient for publication of this case report and any accompanying images. A copy of the written consent is available for review by the Editor-in-Chief of this journal.

## Competing interests

The authors declare that they have no competing interests.

## Authors' contributions

All authors read and approved the final manuscript.
